# Impact of Hypertensive Disorders of Pregnancy on the Risk of Stroke Stratified by Subtypes and Follow-Up Time

**DOI:** 10.1161/STROKEAHA.121.034109

**Published:** 2022-01-05

**Authors:** Shih-Kai Hung, Moon-Sing Lee, Hon-Yi Lin, Liang-Cheng Chen, Chi-Jou Chuang, Chia-Hui Chew, Ben-Hui Yu, Hsuan-Ju Yang, Feng-Chun Hsu, Wen-Yen Chiou

**Affiliations:** Department of Radiation Oncology, Dalin Tzu Chi Hospital, Buddhist Tzu Chi Medical Foundation, Chiayi, Taiwan (S.-K.H., M.-S.L., H.-Y.L., L.-C.C., C.-H.C., B.-H.Y., H.-J.Y., F.-C.H., W.-Y.C.).; School of Medicine, Tzu Chi University, Hualien, Taiwan (S.-K.H., M.-S.L., H.-Y.L., C.-J.C., W.-Y.C.).; Department of Obstetrics and Gynecology, Dalin Tzu Chi Hospital, Buddhist Tzu Chi Medical Foundation, Chiayi, Taiwan (C.-J.C.).

**Keywords:** gestational hypertension, hemorrhagic stroke, hypertension, ischemic stroke, preeclampsia, pregnancy, stroke

## Abstract

**Background and Purpose:**

Hypertensive disorders of pregnancy (HDP) comprise 4 subtypes. Previous studies have not investigated the relationship between stroke risk, different HDP subtypes, and follow-up time, which was the purpose of this study.

**Methods:**

Data of 17 588 women aged 18 to 45 years who had a history of HDP in Taiwan from 2000 to 2017 was retrospectively reviewed. After matching with confounders, 13 617 HDP women and 54 468 non-HDP women were recruited.

**Results:**

HDP women had an adjusted hazard ratio (aHR) of 1.71 (95% CI, 1.46−2.00) for stroke, and 1.60 (1.35−1.89) and 2.98 (2.13−4.18) for ischemic and hemorrhagic stroke, respectively (*P*<0.001 for all). The overall stroke risk in the HDP group was still 2.04 times 10 to 15 years after childbirth (1.47−2.83, *P*<0.001). Although the risks of both ischemic and hemorrhagic stroke persisted, their risk time trends were different. The risk of ischemic stroke reached peak during 1 to 3 years after childbirth with an aHR of 2.14 (1.36–3.38), while hemorrhagic stroke risk gradually increased and had an aHR of 4.64 (2.47−8.73) after 10 to 15 years of childbirth (both *P*<0.001). Among the 4 HDP subtypes, chronic hypertension with superimposed preeclampsia had the highest stroke risk (aHR=3.86, 1.91−7.82, *P*<0.001), followed by preeclampsia–eclampsia (aHR=2.00, 1.63−2.45, *P*<0.001), and gestational hypertension (aHR=1.68, 1.13−2.52, *P*<0.05); chronic preexisting hypertension had the lowest stroke risk (aHR=1.27, 0.97−1.68, *P*>0.05). Furthermore, multiple HDP combined with preeclampsia had aHR of 5.48 (1.14−26.42, *P*<0.05).

**Conclusions:**

The effect of HDP on the risk of future stroke persisted for up to 17 years, both for ischemic and hemorrhagic strokes. The presence of multiple HDP and preeclampsia further increase the stroke risk.

## Abstract 2

Supplemental Digital Content is available in the text.

Approximately 5% to 10% of pregnancies are complicated by hypertensive disorders of pregnancy (HDP),^1,2^ with an incidence of around 15.83 million worldwide in 2017.^3^ HDP is divided into 4 subtypes: chronic hypertension (HTN), chronic HTN with superimposed preeclampsia, preeclampsia/eclampsia, and gestational HTN.^4^ The pathogenesis of HDP remains unclear,^5^ and the etiologies of the 4 HDP subtypes may differ. It is likely that the cause of HDP is multifactorial and involves both genetic and other factors.^6^ One of the hypothesis is insulin resistance, a hallmark of pregnancy which peaks in the third trimester and rapidly returns to prepregnancy levels after delivery^7^; it may play a role in HDP and has been linked to essential HTN.^5^ Its associated conditions, such as gestational diabetes, hyperlipidemia, and obesity, may also predispose to hypertensive pregnancy.^5^ On the other hand, placental ischemia, very-low-density lipoprotein toxicity, immune maladaptation, and genetic imprinting, have also been reported as possible etiologies of preeclampsia.^8^

With these different possible underlying etiologies, it is of interest to investigate whether different HDP subtypes have different patterns of later vascular morbidities. A review of the literature showed that a questionnaire survey study reported that women with a history of HTN during pregnancy, compared with those without such a history, were at an increased risk for the subsequent diagnoses of stroke (12% estimated event rate versus 5%, *P*<0.001).^9^ Another case-control study showed that previous HDP was a predictor for cerebrovascular events with an odds ratio of 4.2.^10^ Among the different HDP subtypes, preeclampsia-eclampsia, as the most studied subtype, was reported to have a relative risk of 1.81 (95% CI, 1.45−2.27) for stroke after 10 years follow-up in a meta-analysis.^11^ However, till date, no study has ever investigated the relationship between the 4 HDP subtypes and the 2 stroke subtypes, namely hemorrhagic and ischemic strokes.

In this 17-year follow-up nationwide cohort study, we evaluated the hemorrhage and ischemic stroke risks in HDP women stratified by the duration of follow-up. Moreover, we incorporated chronic HTN noted during follow-up as a time-dependent covariate into the statistical adjustment for stroke risk associated with HDP. Furthermore, we also compared the stroke risks for all the 4 HDP subtypes.

## Methods

### Data Source and Availability

In this study, we used the Taiwan National Health Insurance Research Database (NHIRD). The information contained within the database was released for research purposes by the Health and Welfare Data Science Center (HWDC), Ministry of Health and Welfare, Taiwan. The raw data from the NHIRD is available to the research community; however, the data must be analyzed within the HWDC after the study proposal is approved (https://dep.mohw.gov.tw/dos/np-2497-113.html). The confidentiality assurances were addressed by following the data regulations of the HWDC. The study protocol, analytic methods, and statistical programming codes are available from the corresponding author on reasonable request.

See the Supplemental Material for the STROBE checklist required by the journal.

The NHIRD contains all the records of diagnosis and treatment of approximately 99% patients in Taiwan.^12^ To ensure accuracy of the claims, the National Health Insurance Administration performs quarterly expert reviews on claims filed by each medical institution,^13^ and the information obtained from the NHIRD is considered both complete and accurate.^14^ The period of collection of data of all pregnant women listed in the Taiwan NHIRD ranged from 2000 to 2017.

### Ethics Statement

The Institutional Review Board of Dalin Tzu Chi Hospital of Buddhist Tzu Chi Medical Foundation (approval number, B10402022) approved this study and waived the requirement for written informed consent from the patients involved because NHIRD is a de-identified database.

### Patients and Study Groups

Between 2000 and 2017, a total of 1 243 150 pregnant women registered with the Taiwan NHIRD were followed till termination of the study period (December 31, 2017), with the shortest and longest follow-up durations of 1 and 17 years, respectively. The International Classification of Diseases, Ninth Revision, Clinical Modification codes for pregnancy and delivery were 650, 651, 652, and 653.

**Figure 1.**
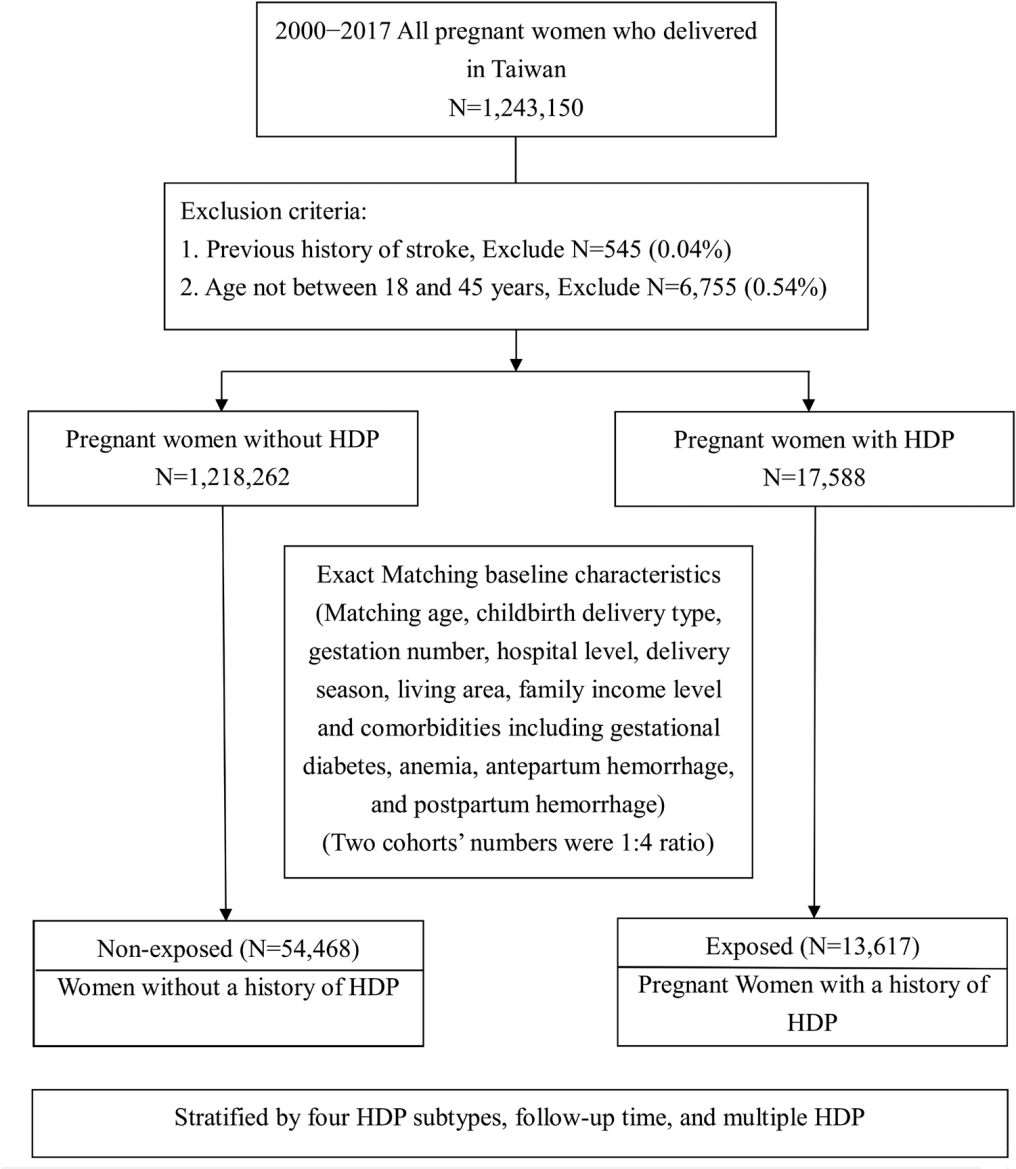
**Flowchart illustrating the selection procedure of the study subjects.** HDP indicates hypertensive disorders of pregnancy.

Figure 1 shows the flow diagram depicting subject enrollment in the present study. We excluded 545 women with a previous history of stroke (0.04%). This study focused on pregnant women aged between 18 and 45 years; therefore, we excluded pregnant women who were below 18 years and above 45 years of age (6755 cases, 0.54%). We included all 4 HDP subtypes (*International Classification of Disease, 9th Revision, Clinical Modification codes 642 and 10th Revision codes* O10−O16). Women with multiple HDP pregnancies were also included in the study. Thus, 1 235 850 women qualified for the analysis.

After exclusion, 1 218 262 women without HDP comprised the non-HDP group, and 17 588 HDP women comprised the HDP group. Because of the large number of women in our nationwide database, we performed exact matching for every HDP subject and control subject. Every woman with HDP had 4 matched control subjects. Every woman and her corresponding matched control subjects had exact the same age, same childbirth delivery type, gestation number, hospital level, delivery season, living area, family income level, and all comorbidities were the same. Comorbidities included chronic HTN, gestational diabetes, anemia, antepartum hemorrhage, and postpartum hemorrhage. Therefore the χ^2^ test for these 2 matched cohorts reveal all baseline characteristics (Table S1) had *P* of 1.000, except HTN noted during follow-up, which was more frequently noted in the HDP group. After exact matching of all these basic characteristics, 13 617 HDP patients and 54 468 non-HDP controls were recruited at a ratio of 1:4.

### Outcome Measures and Potential Confounders

The primary outcome was stroke, which was further divided into hemorrhagic stroke and ischemic stroke. The potential confounders considered in this study were age, type of delivery (cesarean section or normal spontaneous delivery), multiple gestation, multiple HDP, hospital level, season during delivery, comorbidities, and sociodemographic variables (Table 1 and Table S1).

**Table 1.**
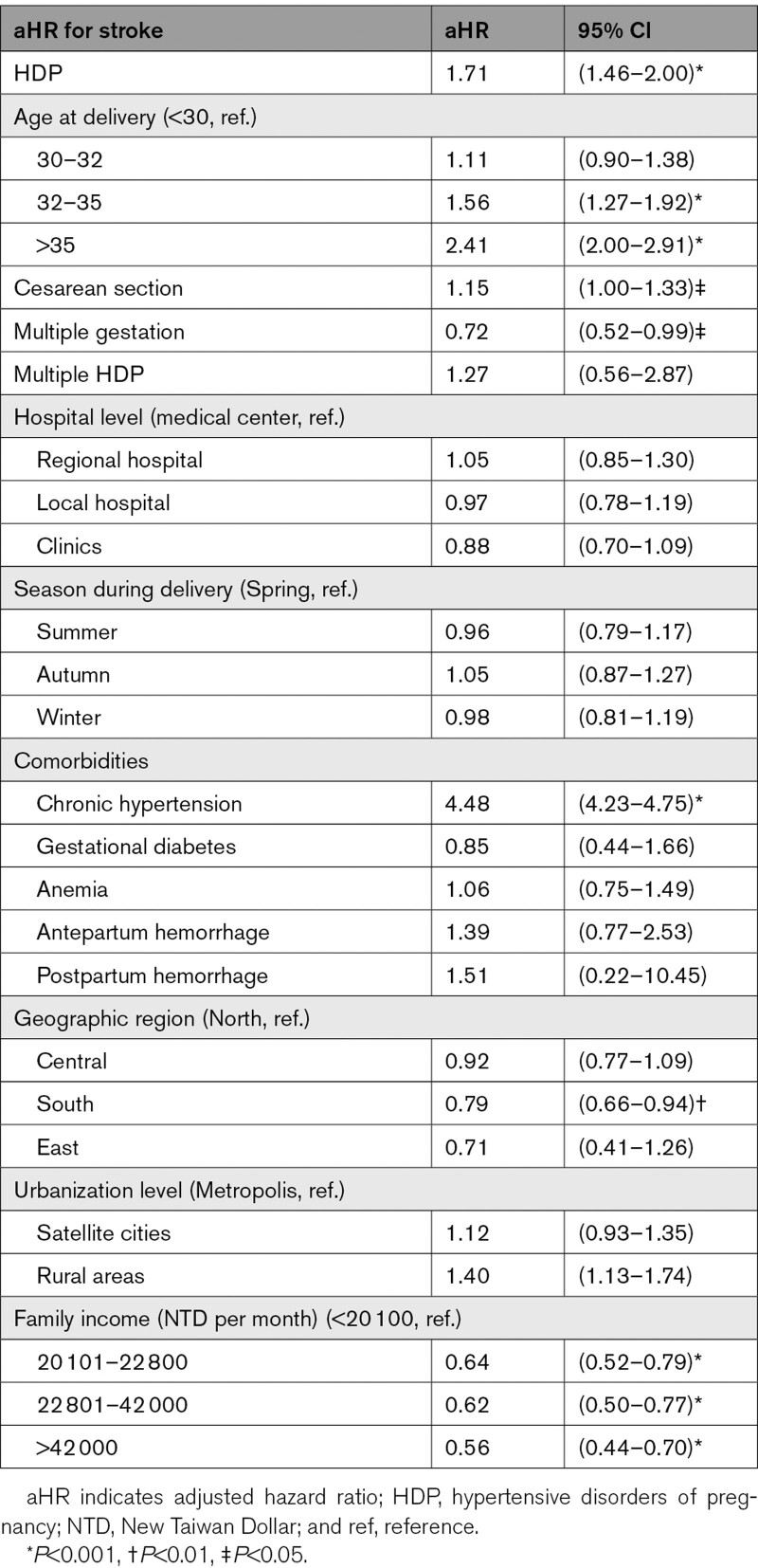
Adjusted Hazard Ratios for Stroke Later in Life After Childbirth in Taiwan, 2000 to 2017

The hospital tiers where the women gave birth included medical centers, regional hospitals, local hospitals, and clinics. Differences in the quality of care during pregnancy and delivery at different hospital levels could affect postpartum recovery and HTN control during pregnancy; thus, the hospital tiers were included in the analysis. The season during delivery was considered a confounding factor because both the weather and seasonal epidemic diseases can affect the intrapartum course.^15,16^

Because this study focused on pregnant women aged between 18−45 years, after matching, none of the women had hyperlipidemia, chronic kidney disease, heart failure, valvular disorders, or peripheral vascular disease in the 2 cohorts.

Lifestyles may differ based on differences in the geographic region, urbanization levels, and family incomes. As patients’ lifestyles may affect the risk of stroke, these sociodemographic factors were adjusted for in this study. The insurance premium for National Health Insurance subscribers in Taiwan is determined according to their family income.

### Statistical Analysis

The basic characteristics between the 2 cohorts comprising women with and without HDP were compared using the χ^2^ test (Table S1).

The incidence of stroke in these 2 cohorts was compared (Table 2). The multivariate Cox regression model was used to calculate the adjusted hazard ratios (aHRs) and 95% CIs for the occurrence of strokes (Table 1). The comorbidities adjusted included chronic HTN noted during follow-up, gestational diabetes, anemia, and delivery conditions such as antepartum hemorrhage and postpartum hemorrhage. All comorbidities were calculated until childbirth except HTN and postpartum hemorrhage. Postpartum hemorrhage was calculated from childbirth until 6 weeks after childbirth.^17^ Because the stroke risk might be affected by chronic HTN after childbirth. Chronic HTN was adjusted in the statistical model and defined as diagnosed at least twice after childbirth and persisting for 6 months or more. Because chronic HTN was calculated until the end of this study, year 2017, chronic HTN was the only one variable adjusted as a time-dependent covariate.

**Table 2.**
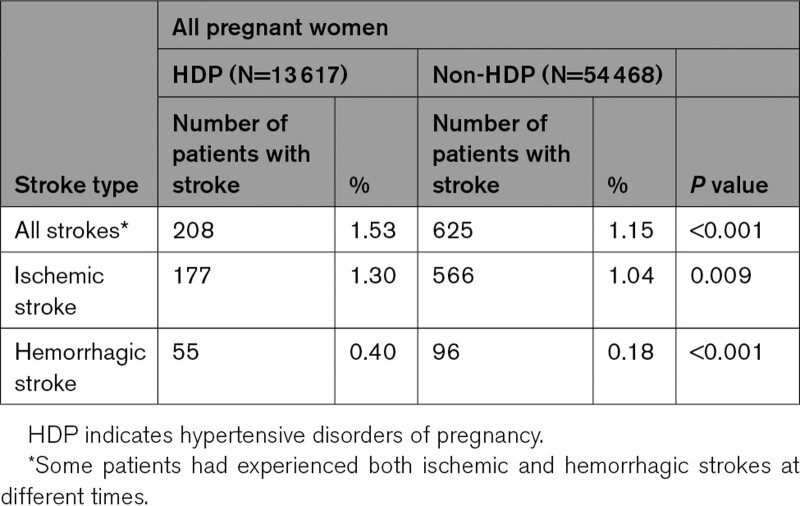
Stroke Prevalence Rate Later in Life in Women With and Without HDP, Stratified by Ischemic Stroke and Hemorrhagic Stroke, in Taiwan, 2000 to 2017

The risk of stroke was further stratified into the risk for ischemic and hemorrhagic stroke (Tables 2 to 4). This study commenced data from the year 2000 to 2017. The follow-up period commenced from the time of delivery and ended on the date of initial stroke diagnosis or till the termination of the study. Owing to the long duration, the follow-up period was divided into short-term (0–1, 1–3, and 3–5 years), intermediate (5–10 years), and long-term (10–15 years) follow ups (Table 3).

**Table 3.**
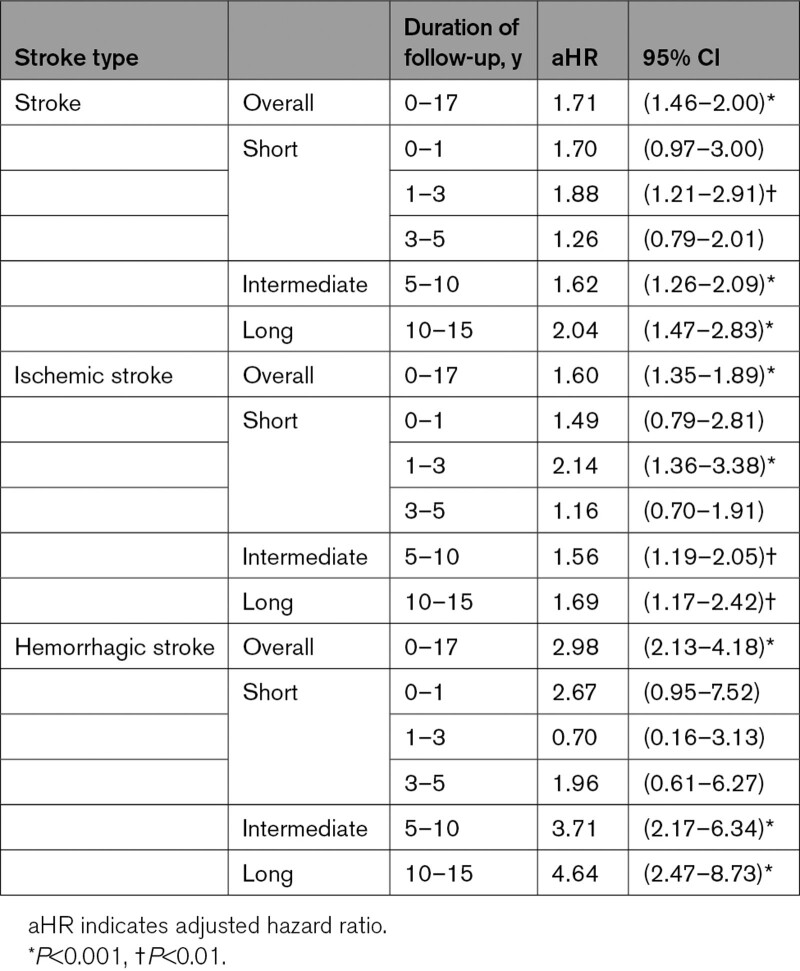
Stroke Risk in Women With a History of Hypertensive Disorders of Pregnancy, Stratified by 2 Stroke Subtypes and Duration of Follow-Up, in Taiwan 2000–2017

In addition to the aforementioned analyses, we also performed stratified analysis for stroke risks of the 4 HDP subtypes and multiple HDP (Table 4).

**Table 4.**
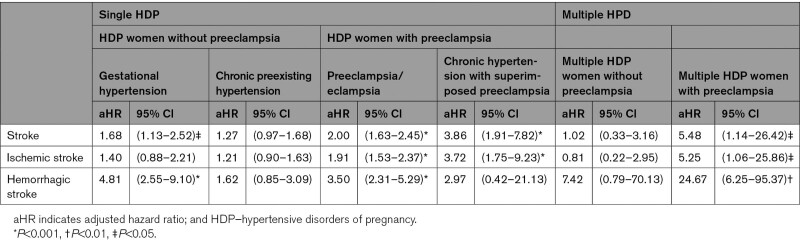
Stroke Risk in Women With a History of HDP, Stratified by HDP Subtypes, and Multiple HDP, in Taiwan 2000 to 2017

**Figure 2.**
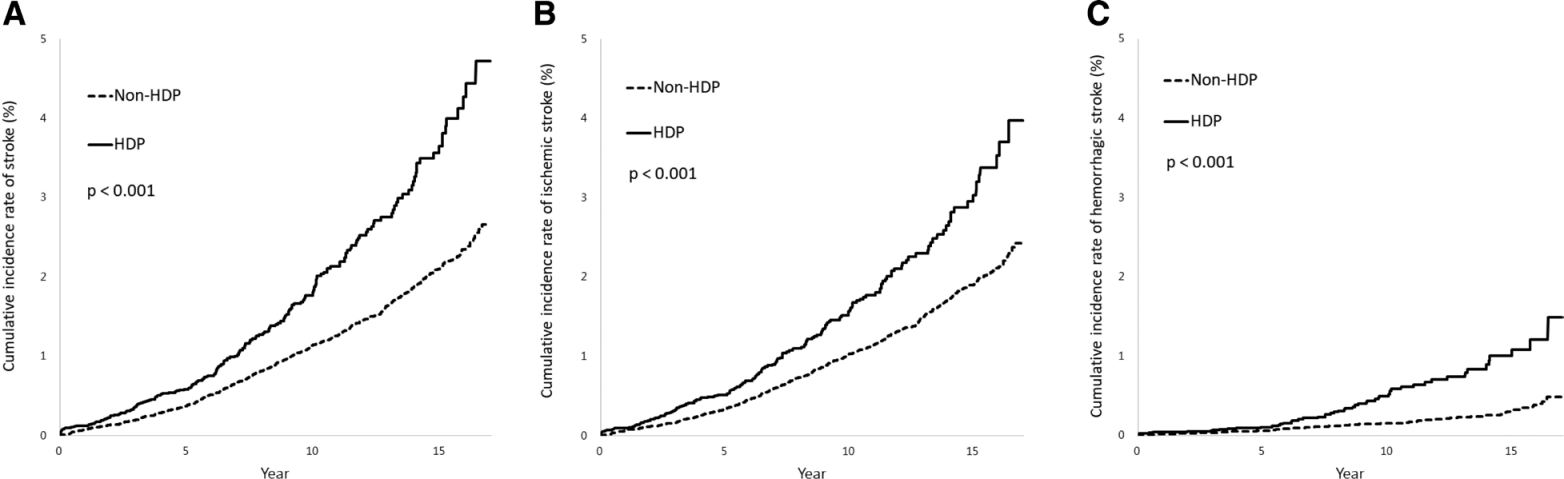
**Cumulative incidence rate of stroke after childbirth in the hypertensive disorders of pregnancy (HDP) and non-HDP groups.** **A**, Overall stroke; (**B**) ischemic stroke; (**C**) hemorrhagic stroke.

Cumulative incidence functions for the occurrence of all strokes, ischemic stroke, and hemorrhagic stroke in women with and without HDP were compared using the Cox proportional-hazards model (Figure 2).

SAS statistical package (version 9.4; SAS Institute, Inc, Cary, NC) was used for all statistical analyses. All tests were 2-sided. A *P*<0.05 was considered statistically significant.

## Results

The mean age of the subjects with and without HDP was 32.14 and 31.92 years, respectively. The baseline distributions of all variables are shown in Table S1. Table 2 shows the prevalence rates of stroke in the study sample. The prevalence rate of all strokes was 1.53% (208 of 13 617) in the HDP group and 1.15% (625 of 54 468) in the non-HDP group (*P*<0.0001).

In the regression model, after adjusting for all confounding variables, HDP significantly increased the stroke risk, with an aHR of 1.71 ([95% CI, 1.46−2.00]; *P*<0.001; Table 1). The age at delivery was also a significant risk for stroke later in life. The aHR for occurrence of stroke was 1.56 for women aged between 32 and 35 years, and 2.41 for those aged >35 years (both *P*<0.001), compared with those aged <30 years at delivery. In this model, HTN noted during follow-up was an obvious risk factor for stroke later in life with an aHR of 4.48 ([95% CI, 4.23−4.75]; *P*<0.001).

The risk of ischemic stroke and hemorrhagic stroke were both significantly higher in the HDP group than in the non-HDP group (aHR, 1.60 [95% CI, 1.35−1.89]; and aHR, 2.98 [95% CI, 2.13−4.18]; both *P*<0.001; Table 3).

### Time Trend of Stroke Risk

The aHRs of overall stroke and stroke subtypes for different follow-up times are listed in Table 3. The risk for overall stroke remained high, with an aHR of 2.04 ([95% CI, 1.47−2.83]; *P*<0.001) after 10 to 15 years follow-up. Table 3 shows that the risk of ischemic stroke peaked during 1 to 3 years after childbirth with an aHR of 2.14 ([95% CI, 1.36−3.38]; *P*<0.001), while hemorrhagic stroke risk gradually increased and had an aHR of 4.64 ([95% CI, 2.47−8.73]; *P*<0.001) after 10 to 15 years.

### The Stroke Risks of the 4 Different HDP Subtypes and Multiple HDP

The stroke risks of the 4 HDP subtypes and multiple HDP are listed in Table 4. Among the 4 HDP subtypes, chronic HTN with superimposed preeclampsia had the highest stroke risk (aHR, 3.86 [1.91−7.82]; *P*<0.001), followed by preeclampsia/eclampsia (aHR, 2.00 [1.63−2.45]; *P*<0.001) and gestational HTN (aHR, 1.68, [1.13−2.52]; *P*<0.05); chronic preexisting HTN had the lowest stroke risk (aHR, 1.27 [0.97−1.68]; *P*>0.05). Multiple HDP combined with preeclampsia had an aHR of 5.48 (1.14−26.42, *P*<0.05).

### Cumulative Incidence Rates

The 17 years cumulative incidence rates of overall strokes, ischemic stroke, and hemorrhagic stroke were higher in the HDP (4.72%, 3.98%, and 1.49%, respectively) than in the non-HDP group (2.66%, 2.43%, and 0.49%, respectively), all with *P* of <0.001, as shown in Figure 2A through 2C).

## Discussion

It is often reported in the literature that HDP increases the risk of stroke. A large cohort study from 1996 to 2001 reported that the relative risks of ischemic stroke and hemorrhagic stroke were 1.29 (1.23–1.35) and 1.14 (1.07–1.21), respectively, in middle-aged UK women with HDP versus those with no such history.^18^ There are many similar studies.^10,18^ Through this long-term follow-up study, we further found that the effect of HDP persisted for 17 years, which was longer than our expectation (Table 3; Figure 2). We also found that although the risks of both ischemic and hemorrhagic strokes persisted, their risk-time trends were different (Table 3). The risk of ischemic stroke peaked around 1 to 3 years post childbirth, while that of hemorrhagic stroke increased after 5 years and kept increasing with time.

Besides, no studies have distinguished between HDP subtypes with and without preeclampsia/eclampsia. The merging of these diseases could lead to bias.^10^ Based on our nationwide database, we found that the stroke risks are higher in HDP subtypes with preeclampsia than those without preeclampsia (Table 4).

A 2019 review study surveying stroke epidemiology in pregnant women reported that the risk of maternal stroke was the highest in the peripartum and early postpartum period.^19^ Most studies investigating the maternal stroke risk during pregnancy reported that preeclampsia may be the major cause.^19^ With regards to the relationship between preeclampsia and stroke, the literature contains many studies.^20–23^ A study reported that although the blood pressure in most preeclampsia women returns to normal within 12 weeks postpartum, the probability of future cerebrovascular disease occurring in women is still much higher than that in the general population.^23^ Our study showed that both multiple HDP and preeclampsia had a high risk for future stroke.

In a short-term study by Tang et al,^24^ the adjusted relative risk of ischemic stroke in preeclampsia group was 4.35 ([95% CI, 0.58−32.92]; *P*>0.05) from 6 to 12 months postpartum. In our stratification study, the aHRs for ischemic stroke was 1.91 in the preeclampsia/eclampsia subtype and 3.72 in the chronic HTN with superimposed preeclampsia subtype, after long-term follow-up (Table 4). In contrast to the Tang’s study which had a short follow-up of 1-year post childbirth, our study of up to 17 years follow-up showed that preeclampsia had a long-term effect on ischemic stroke.

The adjusted relative risk of hemorrhagic stroke in the preeclampsia group was 19.90 (95% CI, 7.75−51.11) from 6 to 12 months postpartum in Tang et al.’s study.^24^ In our study, the aHRs for hemorrhagic stroke in the preeclampsia subtype were 3.50 in the preeclampsia/eclampsia subtype and 2.97 in the chronic HTN with superimposed preeclampsia subtype, after long-term follow-up (Table 4).

A review article on stroke cases during pregnancy and puerperium found that hemorrhagic stroke was more common than ischemic stroke in preeclampsia patients.^25^ Similar to the finding of that study, our study showed that hemorrhagic stroke had a higher aHR than ischemic stroke in 17 years of follow-up period (3.50% versus 1.91%, Table 4).

### Study Strength

This study has several strengths. First, previous studies did not distinguish between HDP subtypes while investigating the relationship between HDP and stroke. Our study differentiated between the 4 HDP subtypes and studied their individual effects. Second, this study reported the differences between ischemic stroke risk and hemorrhagic stroke risk and divided the long follow-up duration into several different follow-up durations to provide information about the time-trend effects of HDP on stroke. Third, the large database, which is likely to be representative of the entire Taiwanese population, provided a good opportunity for exploring the association between HDP and stroke on a nationwide scale. Finally, because of the large number of women in the control group in this nationwide study, we used exact matching to control covariates including socioeconomic status, such as geographic location and income levels, to reduce bias resulting from lifestyle.

### Study Limitation

This study has a limitation. Our data source was the NHIRD, which provides no information on the smoking habits, alcohol consumption, degree of physical activity of patients, or body weight/body mass index. Therefore, we could not evaluate the impact of these factors on stroke outcomes.

### Conclusions

HDP increased the stroke risk, and its effect persisted for up to 17 years. The risk of both ischemic and hemorrhagic strokes persisted; however, their risk time-trends were different. Women with a previous HDP history should be aware that the risk of stroke may persist for even more than a decade and take appropriate precautionary measures or behavior modifications like regular blood pressure monitor, avoiding cigarette smoking and heavy drinking, or preventive medication such as aspirin,^26^ especially for women with multiple HDP combined with preeclampsia.

## Article Information

### Sources of Funding

This study was supported by research grants from the Dalin Tzu Chi Hospital (grant number: DTCRD106-I-09) and the Buddhist Tzu Chi Medical Foundation (TCMF-A 108-06). The funders had no role in the study design, data collection and analysis, decision to publish, or the preparation of the article.

### Disclosures

None.

### Supplemental Material

STROBE Statement

Table S1

## Supplementary Material


