# Supplementary material for: Impact of Hypertensive Disorders of Pregnancy on the Risk of Stroke Stratified by Subtypes and Follow-Up Time
Source: Stroke. 2022 Jan 5;53(2):338–44. doi: 10.1161/STROKEAHA.121.034109 (PMC8785520; doi:10.1161/STROKEAHA.121.034109)
Supplement: Supplementary file 1 [file str-53-338-s001.pdf]

SUPPLEMENTAL MATERIAL

Table S1. Baseline characteristics of pregnant women with and without HDP in Taiwan, 2000-2017

Variables	Women (after 1:4 exact matching)		P-value
	HDP N= 13,617 (%)	Non-HDP N= 54,468 (%)	
Age group of pregnant women			1.000
<30	3,910 (28.71)	15,640 (28.71)	
30−32	3,287 (24.14)	13,148 (24.14)	
33−35	3,139 (23.05)	12,556 (23.05)	
>35	3,281 (24.09)	13,124 (24.09)	
Cesarean section	5,089 (37.37)	20,356 (37.37)	1.000
Multiple gestation	839 (6.16)	3,356 (6.16)	1.000
Multiple HDP	465 (3.41)	NA	NA
Hospital level			1.000
Medical center	2,100 (15.42)	8,400 (15.42)	
Regional hospital	3,335 (24.49)	13,340 (24.49)	
Local hospital	4,472 (32.84)	17,888 (32.84)	
Clinics	3,710 (27.25)	14,840 (27.25)	
Season during delivery			1.000
Spring	3,136 (23.03)	12,544 (23.03)	
Summer	3,313 (24.33)	13,252 (24.33)	
Autumn	3,631 (26.67)	14,524 (26.67)	
Winter	3,537 (25.97)	14,148 (25.97)	
Comorbidities			
Chronic hypertension	2175 (15.97)	1710 (3.14)	<0.001
Gestational diabetes	183 (1.34)	732 (1.34)	1.000
Anemia	801 (5.88)	3204 (5.88)	1.000
Antepartum hemorrhage	160 (1.18)	640 (1.18)	1.000
Postpartum hemorrhage	27 (0.2)	108 (0.2)	1.000
Geographic region			1.000
North	4,589 (33.7)	18,356 (33.7)	
Central	4,614 (33.88)	18,456 (33.88)	
South	4,219 (30.98)	16,876 (30.98)	
East and remote islands	195 (1.43)	780 (1.43)	
Urbanization level			1.000
Metropolis	2582 (18.96)	10328 (18.96)	

Satellite cities	7772 (57.08)	31088 (57.08)	
Rural areas	3263 (23.96)	13052 (23.96)	
Family income (NTD per month)			1.000
1-20,100	1244 (9.14)	4976 (9.14)	
20,101-22,800	5002 (36.73)	20008 (36.73)	
22,801-42,000	4017 (29.5)	16068 (29.5)	
>42,000	3354 (24.63)	13416 (24.63)	

HDP, hypertensive disorders of pregnancy; NTD, New Taiwan Dollar; NA, not applicable.
