# Supplementary figures and images for: Impact of Hypertensive Disorders of Pregnancy on the Risk of Stroke Stratified by Subtypes and Follow-Up Time
Source: Stroke. 2022 Jan 5;53(2):338–44. doi: 10.1161/STROKEAHA.121.034109 (PMC8785520; doi:10.1161/STROKEAHA.121.034109)

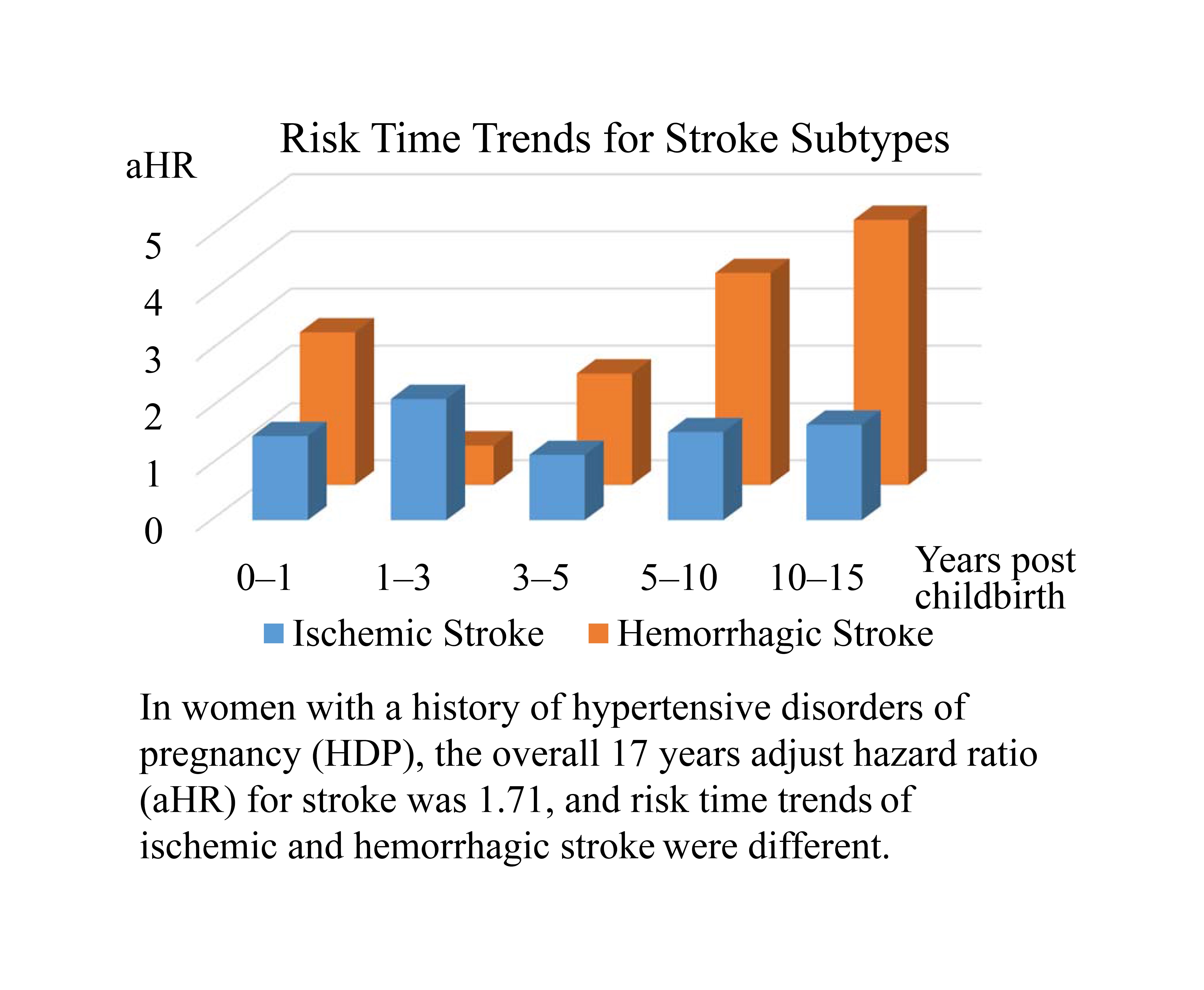

Supplement: Supplementary file 3 [file str-53-338-s003.jpg]
